# Mediterranean Diet: From a Healthy Diet to a Sustainable Dietary Pattern

**DOI:** 10.3389/fnut.2015.00015

**Published:** 2015-05-07

**Authors:** Sandro Dernini, Elliot M. Berry

**Affiliations:** ^1^Forum on Mediterranean Food Cultures, Rome, Italy; ^2^Department of Agriculture and Consumer Protection, Food and Agriculture Organization of the United Nations (FAO), Rome, Italy; ^3^Department of Human Nutrition and Metabolism, Braun School of Public Health, The Hebrew University Hadassah Medical School, Jerusalem, Israel

**Keywords:** Mediterranean diet, sustainable diets, sustainable food systems, food consumption, food cultures, intangible cultural heritage

## Abstract

The notion of the Mediterranean diet has undergone a progressive evolution over the past 60 years, from a healthy dietary pattern to a sustainable dietary pattern, in which nutrition, food, cultures, people, environment, and sustainability all interact into a new model of a sustainable diet. An overview of the historical antecedents and recent increased interest in the Mediterranean diet is presented and challenges related to how to improve the sustainability of the Mediterranean diet are identified. Despite its increasing popularity worldwide, adherence to the Mediterranean diet model is decreasing for multifactorial influences – life styles changes, food globalization, economic, and socio-cultural factors. These changes pose serious threats to the preservation and transmission of the Mediterranean diet heritage to present and future generations. Today’s challenge is to reverse such trends. A greater focus on the Mediterranean diet’s potential as a sustainable dietary pattern, instead than just on its well-documented healthy benefits, can contribute to its enhancement. More cross-disciplinary studies on environmental, economic and socio-cultural, and sustainability dimensions of the Mediterranean diet are foreseen as a critical need.

## Introduction

Mediterranean dietary patterns have developed over the past 5000 or more years spreading from the Fertile Crescent (1) and influenced by the conquests of many different civilizations, the consolidated dietary rules of the three main monotheistic religions (Judaism, Christianity, and Islam), and continuous interactions, additions, and exchanges inside and outside the region. As a result, the Mediterranean diet is an expression of the different food cultures present in the Mediterranean region, with diverse food consumption and production patterns, in continuous evolution representing the particular historical and environmental mosaic that is the Mediterranean (2).

It must be emphasized that there is not one single Mediterranean diet, but rather a number of variations on a basic theme adapted to individual country’s cultures. Therefore, the Mediterranean diet is more than just a defined diet, but it represents the plurality of various cultural expressions of different Mediterranean food cultures and lifestyles.

The term “*Mediterranean diet*” implies the existence of some common dietary characteristics in Mediterranean countries such as: high amounts of olive oil and olives, fruits, vegetables, cereals (mostly unrefined), legumes, and nuts, moderate amounts of fish and dairy products, and low quantities of meat and meat products. Wine in moderation is acceptable when it is not contradictory to religious and social norms (3–5).

The Mediterranean diet has been well-characterized scientifically following the pioneering *Seven Countries Study* conducted by Ancel Keys in the 60s (6). Since then, the Mediterranean diet has been widely studied and reported to be a model of healthy eating associated with significant nutritional and health benefits (7–14).

## Moving Away from the Traditional Mediterranean Diet Model

The Mediterranean area could be described as passing through a “nutritional transition” in which problems of under-nutrition coexist with overweight, obesity, and food-related chronic diseases (15). Under-nutrition is still significant in the South of the Mediterranean: 9.2 million people in 2001–03, 3.9% of the population of the zone, compared with 7.3 million people in 1990–92, 3.8% of the population (16). In 2011, reported rates for overweight and obesity were as follows: 54.4 and 21.3% in Albania; 45.5 and 16.0% in Algeria; 67.9 and 33.1% in Egypt; 50.7 and 18.2% in France; 53.7 and 20.1% in Greece; 54.1 and 19.8% in Italy; 61.8 and 27.4% in Lebanon; 64.3 and 28.8% in Malta; 46.8 and 16.4% in Morocco; 59.1 and 24.0% in Portugal; 62.0 and 26.6% in Spain; 53.7 and 22.3% in Tunisia; and 61.9 and 27.8% in Turkey (17).

Investigations in the early 90s already showed that dietary patterns throughout the Mediterranean countries were increasingly moving away (18–20) from those reported in the 60s. Thus, already in 1995, the Mediterranean diet was considered to be at risk of becoming an “endangered species” (21). Moreover, more recent data have confirmed that in many Mediterranean countries the loss of adherence to the Mediterranean diet is continuing and increasing (22–30), linked also to the current economic downturn (31).

Such a decline in the Mediterranean’s healthy diet patterns was already predicted in 2005 in the Mediterranean Strategy for Sustainable Development report, issued by the United Nations Environment Program, as follows: “*Mediterranean agricultural and rural models, which are at the origins of Mediterranean identity, are under increasing threat from the predominance of imported consumption patterns. This trend is illustrated in particular by the decline of the Mediterranean dietary model despite the recognized positive effects on health*” (32).

Since the early 90s, the healthy Mediterranean diet pattern has been popularized using a pyramid representation as a dietary guideline, in which were highlighted graphically the foods to consume daily, weekly, or less frequently (3). Several dietary guidelines for specific Mediterranean populations have been also developed, associated with a pyramidal representation, such as for the Spanish (33), Greek (34), and Italian populations (35, 36). Since then, various dietary scores of adherence to the Mediterranean diet have been published and extensively reviewed in Ref. (37–41).

**Figure 1:**
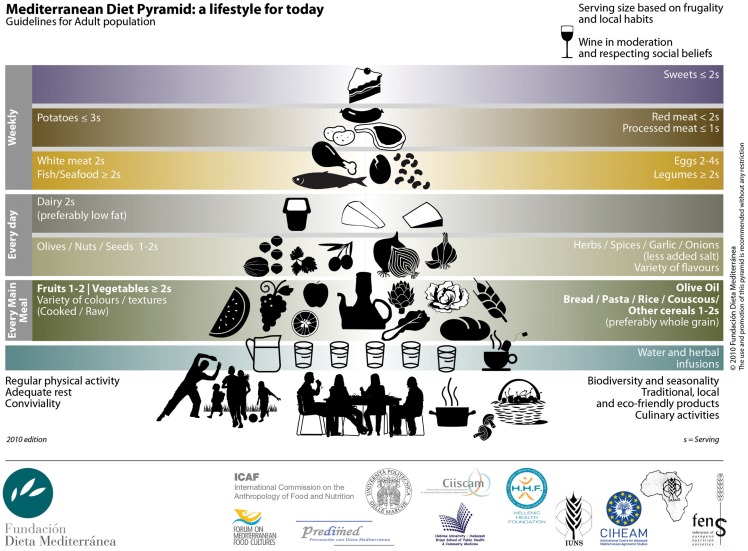
**Mediterranean diet pyramid: a lifestyle for today (5)**.

In 2009 and 2010, through an international scientific consensus process, a new revised Mediterranean diet pyramid (Figure 1) was developed to be adapted to contemporary lifestyles. The new revised Mediterranean diet pyramid was conceived as a simplified main frame to be adapted to different countries specific variations related to the various geographical, socio-economic, and cultural contexts of the contemporary Mediterranean lifestyle, taking into account also their different portions and serving sizes. Daily main meals were highlighted; the concept of frugality and moderation was more emphasized because of the major public health challenge of obesity. In this revised Mediterranean diet pyramid, for the first time, nutrition, eco-friendly products, biodiversity, fruits, and vegetables with a variety of colors, local food production, and conviviality, were brought together with the concept of sustainability (5, 42).

## An Overview of the Themes Surrounding the Mediterranean Diet

Historically, starting from the 1960s, the Mediterranean diet began to be studied as a model of a healthy diet with reduced morbidity and mortality.

Then, in the early 1990s, the Mediterranean diet as a plant-centered diet, consequently lowered demand on soil, water, and energy resources, began to be researched by Joan Dye Gussow as a sustainable dietary pattern, which also considers the overall impact on the ecosystem (43).

In the last decade, the Mediterranean diet has become the object of increasing studies on its environmental sustainability, because of its mainly plant-based dietary pattern and its lower greenhouse gas emissions and lower water footprints, when compared to current Western dietary patterns (44–55).

In 2009, the international conference “The Mediterranean diet as a sustainable diet model” was organized in Parma, Italy, by the International Interuniversity Studies Center on Mediterranean Food Cultures (CIISCAM), with the technical collaboration of FAO, the Italian National Institute of Food and Nutrition (INRAN), and the International Center for Advanced Mediterranean Agronomic Studies (CIHEAM) of Bari. The CIHEAM is an intergovernmental organization composed of ministers of agriculture of its 13 member states. At this conference, the Mediterranean diet was analyzed as a sustainable diet model, because of its nutritional, environmental, economic, and socio-cultural dimensions at the core of the sustainability rationale (56). On this occasion, an international consensus was also reached on a new revised Mediterranean diet pyramid in which, for the first time, biodiversity and eco-friendly products, with a lower impact on the environment, were inserted together with main Mediterranean diet characteristic foods (5, 42).

As a follow up to it, in 2010, FAO and Bioversity International organized in collaboration with CIHEAM-Bari and INRAN, an international scientific symposium on “biodiversity and sustainable diets,” in which a consensus position was reached on a definition of “sustainable diets,” as follows: “*Sustainable diets are those diets with low environmental impacts which contribute to food and nutrition security and to healthy life for present and future generations. Sustainable diets are protective and respectful of biodiversity and ecosystems, culturally acceptable, accessible, economically fair and affordable; nutritionally adequate, safe and healthy; while optimizing natural and human resources*” (57). Within this definition, on this occasion, the Mediterranean diet was acknowledged as a sustainable diet example. Therefore, FAO and CIHEAM-Bari started a joint collaboration on the Mediterranean diet as a case study on which to develop and validate methods and indicators for the assessment of the sustainability of diets and food consumption patterns in the Mediterranean area. In line with the definition, four main thematic areas were identified: (1) nutrition, health, and lifestyle; (2) environment including agro-biodiversity; (3) economy; (4) society and culture (58, 59).

**Figure 2:**
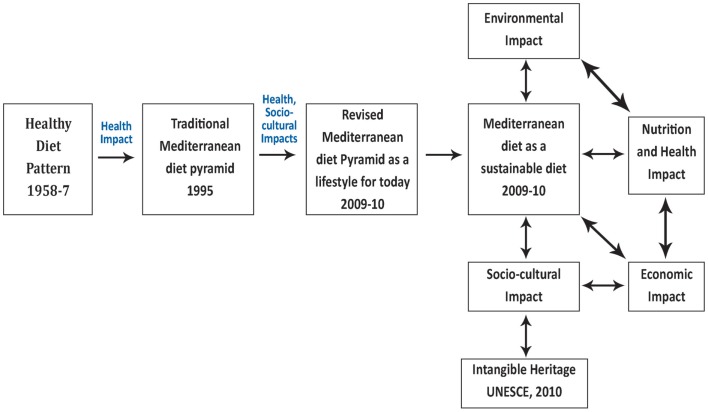
**The evolution of concepts surrounding the Mediterranean diet (2)**.

At the end of 2010, the Mediterranean diet was inscribed in the UNESCO Representative List of the Intangible Cultural Heritage of Humanity, and described as follows: *“The Mediterranean Diet – derived from the Greek word díaita, way of life – is the set of skills, knowledge, rituals, symbols, and traditions, ranging from the landscape to the table, which in the Mediterranean basin concerns the crops, harvesting, picking, fishing, animal husbandry, conservation, processing, cooking, and particularly sharing and consuming of food”* (60) (Figure 2). This UNESCO acknowledgment highlighted that *the Mediterranean diet notion was encompassing a social cultural expression of the different food cultures of the Mediterranean* and the importance of the Mediterranean diet was not just in its specific foods and nutrients, but in the way in which its characteristic foods were produced, cooked, and eaten.

From 2011 to 2012, as outcome of the FAO/CIHEAM case study on the sustainability of the Mediterranean diet, through a participatory joint process, conducted with the FAO/UNEP Sustainable Food Systems (SFSs) Program in collaboration with the Forum on Mediterranean Food Cultures, CIISCAM/Sapienza University of Rome, ENEA, CNR, INRAN, Bioversity International, and WWF-Italy, a first outline of a methodological approach was developed for the FAO/CIHEAM discussion paper “Toward the Development of Guidelines for Improving the Sustainability of Diets and Food Consumption Patterns in the Mediterranean Area” and presented within the international seminar “The Sustainability of Food Systems in the Mediterranean Area,” jointly organized on the occasion of the 9th Meeting of the CIHEAM Ministers of Agriculture, held in 2012 in Malta (58). This discussion paper also provided a first list of potential sustainability indicators, based on existing data source, taking into account priority challenges (58, 59). In the final CIHEAM Ministers’ declaration, the role of the Mediterranean diet was underlined *“as a driver of SFS within the strategies of regional development and on that of traditional local products, since quantitative food security must also be complemented by qualitative approaches*,” and it was also recommended to support the implementation of the outcomes of the FAO/CIHEAM International Seminar (58).

As a follow up, CIHEAM-Bari started in 2013 a pilot project on “Evaluation and valorization of the sustainability of quality products of Apulia, Italy.” Its objective was to apply the methodological approach jointly developed with FAO to a well-defined territorial context, such as the Apulia region (61), to add together, environmental, economic, nutritional-health, and socio-cultural sustainable values to local quality typical products, which are also the cornerstone of the regional Mediterranean diet and food system. This methodological approach was then further developed in a White Paper 5 “*Mediterranean food consumption patterns: diet, environment, society, economy, and health*” prepared by CIHEAM-Bari and FAO-SFS Program for *Feeding Knowledge project* in view of EXPO 2015 in Milan (62).

## Discussion

One of the most important challenges faced especially by southern and eastern Mediterranean countries is food and nutrition security (63), in which problems of under-nutrition coexist, within a “nutritional transition” process, with overweight, obesity, and food-related chronic diseases (15). Population growth, globalization, urbanization, and socio-economic factors are causing changes in diets and consumption patterns in the Mediterranean region and southern European countries (64–68).

Today, a main concern for the Mediterranean food and agricultural sector is also to conserve natural resources for future generations while providing simultaneously enough food, in quantity and quality, to meet the nutritional needs of a growing population. Therefore, changes toward optimizing both food consumption and food production are foreseen to ensure more SFSs and contribute to achieve food and nutrition security in the Mediterranean region (62).

Food security, nutrition, and sustainability have been increasingly discussed in the same context (69–72). The High Level Panel of Experts on Food Security and Nutrition (HLPE) has recently provided the following definitions for SFS and for food system: “*A SFS is a food system that delivers food security and nutrition for all in such a way that the economic, social, and environmental bases to generate food security and nutrition for future generations are not compromised; A food system gathers all the elements (environment, people, inputs, processes, infrastructures, institutions, etc.) and activities that relate to the production, processing, distribution, preparation, and consumption of food and the outputs of these activities, including socio-economic and environmental outcomes*” (72).

Food systems around the world are changing rapidly, with profound implications for diets and food consumption outcomes. Food consumption is variably affected by a wide range of factors including food availability, food accessibility, and food choices, which in turn may be influenced by geography, demography, disposable income, socio-economic status, urbanization, globalization, religion, culture, marketing, and consumer attitude (73, 74).

Consumer choice can play a leading role to orient food production toward sustainability, by selecting certain types of products according to their geographic origin, production process, thereby creating value especially for small producers. “Sustainable consumption and production” recognizes the role of consumers to promote sustainability, and sustainable production, by their consumption choices. To increase the sustainability of food systems, both production and consumption and supply and demand, have to be considered (75).

Food consumption trends and patterns have been considered among the most important drivers of environmental pressures (74, 76). Sustainable diets are considered those diets that have *“low environmental impact and are respectful of biodiversity while optimizing natural and human resources*” (57). The Mediterranean diet model in many studies has been appreciated to have a lower environmental impact, mainly due to its consumption of more plant-derived products and less animal products, with respect to other current dietary patterns (44–54). A recent study has shown that a better adherence of the Spanish population toward the Mediterranean diet pattern would have reduced greenhouse gas emissions, land use, and energy consumption and to a lower extent water consumption (55).

Some studies have shown that foods with lower greenhouse gas emissions do not have always also higher nutritional values (77, 78), and more studies are also needed to assess the relation between the nutrient adequacy of individual foods and total diets in relation to multiple sustainability assessments (79).

The assessment of the sustainability of the Mediterranean diet in different countries requires also to evaluate interactions and correlations, direct and indirect, between nutrition/health and environmental indicators and indicators related to the socio-cultural and economic dimensions of sustainability. Because all current adherence scores refer only to the nutritional and health benefits of the traditional Mediterranean diet model (37–41), there is also a need to develop and validate new comprehensive adherence scores for the Mediterranean diet model as a sustainable dietary pattern for the contemporary times. The centrality of the individual, the consumer, should be also considered in the assessment of the sustainability of the Mediterranean diet, in spite of lack of data availability on individuals in most of the southern and eastern Mediterranean countries.

## Conclusion

Numerous questions still need to be addressed on the broader concept of the sustainability of the Mediterranean diet, particularly after its acknowledgment by UNESCO as an intangible cultural heritage, with a need for providing more assessments of its socio-cultural and economic sustainability, which are still lacking.

Food plays a central role in the social and cultural life of the Mediterranean area. In this context, the Mediterranean diet is a complex web of inter-related cultural aspects, and it must always be considered as a part of significant social and cultural interdependent Mediterranean food systems, and never as an independent item (80, 81). Therefore, a SFSs approach, in which also its socio-cultural and economic benefits are highlighted together with the well-appreciated healthy and environmental ones, can contribute to the enhancement of the Mediterranean diet.

More interdisciplinary studies and country-based approaches need to be developed, in the context of Mediterranean SFSs improvement, to better understand potential interactions between the Mediterranean diet, and the sustainability of the food consumption and production of its characteristic foods.

The ongoing CIHEAM/FAO case study, as part of the further development of the FAO/UNEP SFSs program, within the 10-Year Framework of Programs on Sustainable Consumption and Production (82), will provide more understanding on linkages between the Mediterranean diet and Mediterranean food systems. This shift of interest on the Mediterranean diet, from a healthy diet to a sustainable dietary pattern, would also contribute to the improvement of the sustainability of Mediterranean food systems and food security and nutrition in the Mediterranean area.

A greater focus on the Mediterranean diet’s potential as a sustainable dietary pattern would be beneficial for its revitalization. Therefore, more cross-cutting studies through the nutrition/health, environmental, economic, and socio-cultural sustainability dimensions of the Mediterranean diet are foreseen as a critical need for it.

## Conflict of Interest Statement

The authors declare that the research was conducted in the absence of any commercial or financial relationships that could be construed as a potential conflict of interest.

## References

[1] Berry E Arnoni Y Aviram M The middle eastern & biblical origins of the Mediterranean diet. Public Health Nutr (2011) 14(12A):2288–95. 10.1017/S1368980011002539 22166186

[2] Dernini S Berry EM Historical and behavioral perspectives of the Mediterranean diet. In: Romagnolo DF, editor. Mediterranean Diet in Health and Disease. London: Springer Press (2015). (in press).

[3] Willett W Sacks F Trichopoulou A Drescher G Ferro-Luzzi A Helsing E Mediterranean diet pyramid: a cultural model for healthy eating. Am J Clin Nutr (1995) 61:1402S–6S. 7754995 10.1093/ajcn/61.6.1402S

[4] Trichopoulou A Lagiou P. Healthy traditional Mediterranean diet: an expression of culture, history and lifestyle. Nutr Rev (1997) 55:383–9. 10.1111/j.1753-4887.1997.tb01578.x 9420448

[5] Bach-Faig A Berry EM Lairon D Reguant J Trichopoulou A Dernini S Mediterranean diet pyramid today. Science and cultural updates. Public Health Nutr (2011) 14(12A):2274–84. 10.1017/S1368980011002515 22166184

[6] Keys A Coronary heart disease in seven countries. Circulation (1970) 41(Suppl I):211. 5442775

[7] Estruch R Ros E Salas-Salvadó J Covas MI Corell D Arós F Primary prevention of cardiovascular disease with a Mediterranean diet. N Engl J Med (2013) 368:1279–90. 10.1056/NEJMoa1200303 29897867

[8] Kastorini CM Milionis HJ Esposito K Giugliano D Goudevenos JA Panagiotakos DB. The effect of Mediterranean diet on metabolic syndrome and its components a meta-analysis of 50 studies and 534,906 individuals. J Am Coll Cardiol (2011) 57(11):1299–313. 10.1016/j.jacc.2010.09.073 21392646

[9] Martínez-González MA Bes-Rastrollo M Serra-Majem L Lairon D Estruch R Trichopoulou A. Mediterranean food pattern and the primary prevention of chronic disease: recent developments. Nutr Rev (2009) 67(Suppl 1):S111–6. 10.1111/j.1753-4887.2009.00172.x 19453663

[10] Serra-Majem L Roman B Estruch R. Scientific evidence of interventions using the Mediterranean diet: a systematic review. Nutr Rev (2006) 64:S27–47. 10.1301/nr.2006.feb.S27-S47 16532897

[11] Bosetti C Pelucchi C La Vecchia C. Diet and cancer in Mediterranean countries: carbohydrates and fats. Public Health Nutr (2009) 12(9A):1595–600. 10.1017/S1368980009990425 19689827

[12] Maillot M Issa C Vieux F Lairon D Darmon N. The shortest way to reach nutritional goals is to adopt Mediterranean food choices. Evidence from computer-generated personalized diets. Am J Clin Nutr (2011) 94(4):1127–37. 10.3945/ajcn.111.016501 21900460

[13] Buckland G Bach A Serra-Majem L. Obesity and the Mediterranean diet: a systematic review of observational and intervention studies. Obes Rev (2008) 9:582–93. 10.1111/j.1467-789X.2008.00503.x 18547378

[14] Sofi F Cesari F Abbate R Gensini GF Casini A. Adherence to Mediterranean diet and health status: meta-analysis. BMJ (2008) 337:a1344. 10.1136/bmj.a1344 18786971 PMC2533524

[15] Belahsen R. Cultural diversity of sustainable diets. Nutrition transition and food sustainability. Proc Nutr Soc (2014) 73:385–8. 10.1002/jsfa.6351 24824339

[16] CIHEAM. MediTerra. The Future of Agriculture and Food in Mediterranean Countries. Paris: CIHEAM–SciencesPo Les Presses (2008).

[17] WHO. Non Communicable Diseases Country Profiles 2011. Global Report. (2011). Available from: http://www.who.int/nmh/publications/ncd_profiles2011/en/index.html

[18] Kafatos AI Kouroumalis I Vlachonikolis I Theodorou C Labadarios D. Coronary-heart-disease risk-factor status of the Cretan urban population in the 1980s. Am J Clin Nutr (1991) 54:591–8. 1877515 10.1093/ajcn/54.3.591

[19] Alberti-Fidanza A Fidanza F Chiuchiu M Verducci G Fruttini D. Dietary studies on two rural Italian population groups of the Seven Countries Study. 3. Trend of food and nutrient intake from 1960 to 1991. Eur J Clin Nutr (1999) 53:854–60. 10.1038/sj.ejcn.1600865 10556997

[20] Serra-Majem L Helsing E, editors. Changing patterns of fat intake in Mediterranean countries. Eur J Clin Nutr (1993) 47(Suppl):1. 8269891

[21] Nestle M. Mediterranean diets: historical and research overview. Am J Clin Nutr (1995) 61(Suppl):1313S–20S. 7754981 10.1093/ajcn/61.6.1313S

[22] Garcia-Closas R Berenguer A Gonzalez C. Changes in food supply in Mediterranean countries from 1961 to 2001. Public Health Nutr (2006) 9(1):53–60. 10.1079/PHN2005757 16480534

[23] Belahsen R Rguibi M. Population health and Mediterranean diet in southern Mediterranean countries. Public Health Nutr (2006) 9(8A):1130–5. 10.1017/S1368980007668517 17378952

[24] Alexandratos N The Mediterranean diet in a world context. Public Health Nutr (2006) 9(1A):111–7. 10.1079/PHN2005932 16512957

[25] da Silva R Bach-Faig A Raido Quintana B Buckland G Vaz de Almeida MD Serra-Majem L. World variation of adherence to the Mediterranean diet, in 1961-1965 and 2000-2003. Public Health Nutr (2009) 12(9A):1676–84. 10.1017/S1368980009990541 19689839

[26] Varela-Moreiras G Avila JM Cuadrado C del Pozo S Ruiz E Moreiras O. Evaluation of food consumption and dietary patterns in Spain by the food consumption survey: updated information. Eur J Clin Nutr (2010) 64:S37–43. 10.1038/ejcn.2010.208 21045847

[27] Vareiro D Bach-Faig A Raidó Quintana B Bertomeu I Buckland G Vaz de Almeida MD Availability of Mediterranean and non-Mediterranean foods during the last four decades: comparison of several geographical areas. Public Health Nutr (2009) 12(9A):1667–75. 10.1017/S136898000999053X 19689838

[28] Aounallah-Skhiri H Traissac P El Ati J Eymard-Duvernay S Landais E Achour N Nutrition transition among adolescents of a south-Mediterranean country: dietary patterns, association with socio-economic factors, overweight and blood pressure. A cross-sectional study in Tunisia. Nutr J (2011) 10:38 10.1186/1475-2891-10-38 21513570 PMC3098773

[29] León-Muñoz LM Guallar-Castillón P Graciani A López-García E Mesas AE Aguilera MT Adherence to the Mediterranean diet pattern has declined in Spanish adults. J Nutr (2012) 142(10):1843–50. 10.3945/jn.112.164616 22875552

[30] Roccaldo R Censi L D’Addezio L Toti E Martone D D’Addesa D Adherence to the Mediterranean diet in Italian school children (The ZOOM8 Study). Int J Food Sci Nutr (2014) 65(5):621–8. 10.3109/09637486.2013.873887 24527679

[31] Bonaccio M Di Castelnuovo A Bonanni A Costanzo S De Lucia F Persichillo M Decline of the Mediterranean diet at a time of economic crisis. Results from the Moli-sani study. Nutr Metab Cardiovasc Dis (2014) 24(8):853–60. 10.1016/j.numecd.2014.02.014 24819818

[32] UNEP/MAP. Mediterranean strategy for sustainable development: a framework for environmental sustainability and shared prosperity. Tenth Meeting of the Mediterranean Commission on Sustainable Development (MCSD). Athens (2005). Available from: http://www.un.org/esa/sustdev/natlinfo/indicators/egmIndicators/MSSD_latest_eng.pdf

[33] Aranceta J Serra-Majem L. Dietary guidelines for the Spanish population. On behalf of the working party for the development of food-based dietary guidelines for the Spanish population. Public Health Nutr (2001) 4(6A):1403–8. 10.1079/PHN2001228 11918490

[34] Supreme Scientific Health Council Ministry of Health and Welfare of Greece. Dietary guidelines for adults in Greece. Arch Hellenic Med (1999) 16:516–24.

[35] Calabrese G Cannella C Carruba M Morino GS Spera G On Behalf of the Working Expert Group of the Italian Health Ministry to Orient the Citizen Towards Healthier Behaviors. Rome: Ministero della Salute (2004).

[36] del Balzo V Diolordi L Pinto A Giusti AM Vitiello V Cannella C Mediterranean diet pyramids: towards the Italian model. Ann Ig (2012) 24:443–7. 23193900

[37] Bach A Serra-Majem L Carrasco JL Roman B Ngo J Bertomeu I The use of indexes evaluating the adherence to the Mediterranean diet in epidemiological studies: a review. Public Health Nutr (2006) 9(1A):132–46. 10.1079/PHN2005936 16512961

[38] Waijers P Feskens E Ocke M. A critical review of predefined quality scores. Br J Nutr (2007) 97:219–31. 10.1017/S0007114507250421 17298689

[39] Kourlaba G Panagiotakos DB. Dietary quality indices and human health: a review. Maturitas (2009) 62:1–8. 10.1016/j.maturitas.2008.11.021 19128905

[40] Milà-Villarroel R Bach-Faig A Puig J Puchal A Farran A Serra-Majem L Comparison and evaluation of the reliability of indexes of adherence to the Mediterranean diet. Public Health Nutr (2011) 14(2A):2338–45. 10.1017/S1368980011002606 22166193

[41] Sofi F Macchi C Abbate R Gensin GF Casini A. Mediterranean diet and health status: an updated meta-analysis and a proposal for a literature-based adherence score. Public Health Nutr (2013) 17(12):2769–82. 10.1017/S1368980013003169 24476641 PMC10282340

[42] Dernini S Berry EM Bach-Faig A Belahsen R Donini LM Lairon D A dietary model constructed by scientists: the Mediterranean diet. In: Mombiela F, editor. Mediterra 2012: The Mediterranean Diet for Sustainable Regional Development. Paris: CIHEAM-SciencesPo Les Presses (2012). p. 71–88.

[43] Gussow JD. Mediterranean diets: are they environmentally responsible? Am J Clin Nutr (1995) 61(Suppl):1383S–9S. 7754992 10.1093/ajcn/61.6.1383S

[44] Tilman D Clark M. Global diets link environmental sustainability and human health. Nature (2014) 515:518–22. 10.1038/nature13959 25383533

[45] van Dooren C Marinussen M Blonk H Aiking H Vellinga P Exploring dietary guidelines based on ecological and nutritional values: a comparison of six dietary patterns. Food Policy (2014) 44:36–46. 10.1016/j.foodpol.2013.11.002

[46] Pairotti MB Cerutti AK Martini F Vesce E Padovan D R B. Energy consumption and GHG emission of the Mediterranean diet: a systemic assessment using a hybrid LCA-IO method. J Clean Prod (2014) 1–10. 10.1016/j.jclepro.2013.12.082

[47] Capone R Iannetta M El Bilali H Colonna N Debs P Dernini S A preliminary assessment of the environmental sustainability of the current Italian dietary pattern: water footprint related food consumption. J Food Nutr Res (2013) 1(4):59–67. 10.12691/jfnr-1-4-5

[48] Tukker A Goldbohm RA de Koning A Verheijden M Kleijn R Wolf O Environmental impact of changes to healthier diets in Europe. Ecol Econ (2011) 70:1776–88. 10.1016/j.ecolecon.2011.05.001

[49] Barilla Center for Food and Nutrition. Double Pyramid: Healthy Food for People, Sustainable Food for the Planet. Parma (2010). Available from: http://www.barillacfn.com/wp-content/uploads/2010/06/pp_doppia_piramide_alimentazione_eng.pdf

[50] Pluimers J Blonk H Methods for Quantifying the Environmental and Health Impacts of Food Consumption Patterns. Gouda: Blonk Milieu advies (2011).

[51] EC/JRC. Environmental Impacts of Diet Changes in the EU. Technical Report, European Commission (EC). Sevilla: Joint Research Centre (DG JRC), Institute for Prospective Technological Studies (2009).

[52] Baroni L Cenci L Tettamanti M Berati M. Evaluating the environmental impact of various dietary patterns combined with different food production systems. Eur J Clin Nutr (2007) 61(2):279–86. 10.1038/sj.ejcn.1602522 17035955

[53] de Boer J Helms M Aiking H Protein consumption and sustainability: diet diversity in EU-15. Ecol Econ (2006) 59:267–74. 10.1016/j.ecolecon.2005.10.011

[54] Duchin F Sustainable consumption of food: a framework for analyzing scenarios about changes in diets. J Ind Ecol (2005) 9(1–2):99–114. 10.1162/1088198054084707

[55] Almendros SS Obrador B Bach-Faig A Serra-Majem L. Environmental footprints of Mediterranean versus western dietary patterns: beyond the health benefits of the Mediterranean diet. Environ Health (2013) 12(1):118. 10.1186/1476-069X-12-118 24378069 PMC3895675

[56] Burlingame B Dernini S. Sustainable diets: the Mediterranean diet example. Public Health Nutr (2011) 14(12A):2285–7. 10.1017/S1368980011002527 22166185

[57] FAO/Bioversity. In: Burlingame B Dernini S, editors. Sustainable Diets and Biodiversity. Directions and Solutions for Policy, Research and Action. Rome: FAO (2012). p. 7.

[58] FAO/CIHEAM. Towards the Development of Guidelines for Improving the Sustainability of Diets and Food Consumption Patterns in the Mediterranean Area. Rome (2012). Available from: http://www.fao.org/docrep/016/ap101e/ap101e.pdf

[59] Dernini S Meybeck A Burlingame B Gitz G Lacirignola C Debs P Developing a methodological approach for assessing the sustainability of diets: the Mediterranean diet as a case study. New Medit (2013) 12(3):28–36.

[60] UNESCO. Representative List of the Intangible Cultural Heritage of Humanity. Paris (2010). Available from: http://www.unesco.org/culture/ich/RL/00884

[61] Lacirignola C Capone R Bottalico F El Bilali H Debs P Sustainability of typical quality products for food and nutrition security in the Mediterranean: lessons from the case of Apulia region in Italy. In: Feeding Expo Milano with Mediterranean Perspectives. (Vol. 32), Paris: Watch Letter (2015).

[62] CIHEAM/FAO. Mediterranean Food Consumption Patterns: Diet, Environment, Society, Economy and Health. A White Paper Priority 5. Expo Milan 2015 Feeding Knowledge Programme. Rome: CIHEAM-Bari/FAO (2015).

[63] FAO. Regional Priority Framework for the Near East. FAO Regional Office for the Near East. Cairo (2012). Available from: http://www.fao.org/fileadmin/user_upload/rne/docs/RPF-EN.pdf

[64] Plan Bleu. 20 Years of Sustainable Development in the Mediterranean: Review and Outlook. (Vol. 22). Blue Plan Notes (2012). Available from: http://planbleu.org/sites/default/files/publications/8p22_20ans_dd_en.pdf

[65] Padilla M Dietary patterns and trends in consumption. In: Mombiela F, editor. Mediterra 2008: The Future of Agriculture and Food in Mediterranean Countries. Paris: CIHEAM Presses de Sciences Po (2008). p. 149–70.

[66] Florensa S Aragall X Mutations in Mediterranean societies. In: Mombiela F, editor. Mediterra 2012. Paris: CIHEAM-SciencesPo Les Presses (2012). p. 91–113.

[67] González Turmo I The Mediterranean diet: consumption, cuisine and food habits. In: Mombiela F, editor. Mediterra 2012: The Mediterranean Diet for Sustainable Regional Development. Paris: CIHEAM SciencesPo Les Presses (2012). p. 115–32.

[68] Beghin L Dauchet L De Vriendt T Cuenca-Garcıa M Manios Y Toti E Influence of parental socio-economic status on diet quality of European adolescents: results from the HELENA study. Br J Nutr (2014) 111:1303–12. 10.1017/S0007114513003796 24330831

[69] Garnett T. Food sustainability: problems, perspectives and solutions. Proc Nutr Soc (2013) 72:29–39. 10.1017/S0029665112002947 23336559

[70] Lang T Barling D. Nutrition and sustainability: an emerging food policy discourse. Proc Nutr Soc (2013) 72:1–12. 10.1017/S002966511200290X 23217475

[71] Berry EM Dernini S Burlingame B Meybeck A Conforti P. Food security and sustainability: can one exist without the other? Public Health Nutr (2015):1–10. 10.1017/S136898001500021X 25684016 PMC10271846

[72] HLPE. Food Losses and Waste in the Context of Sustainable Food Systems. Report HLPE. Rome (2014). Available from: http://www.fao.org/3/a-i3901e.pdf

[73] Kearney J Food consumption trends and drivers. Philos Trans R Soc Lond B Biol Sci (2010) 365:2793–807. 10.1098/rstb.2010.0149 20713385 PMC2935122

[74] Reisch L Eberle U Lorek S Sustainable food consumption: an overview of contemporary issues and policies. Sustain Sci Pract Policy (2013) 9(2):7–25.

[75] Meybeck A Gitz V Signs to choose: voluntary standards and ecolabels as information tools for consumers. In: Meybeck A Redfern S, editors. Voluntary Standards for Sustainable Food Systems: Challenges and Opportunities. Proceedings of a Joint FAO/UNEP Workshop Rome: FAO (2014). Available from: http://www.fao.org/docrep/019/i3421e/i3421e.pdf

[76] Carlsson-Kanyama A Gonzalez A. Potential contributions of food consumption patterns to climate change. Am J Clin Nutr (2009) 89(5):1704S–9S. 10.3945/ajcn.2009.26736AA 19339402

[77] Macdiarmid JI. Is a healthy diet an environmentally sustainable diet? Proc Nutr Soc (2013) 72:13–20. 10.1017/S0029665112002893 23186839

[78] Vieux F Soler LG Touazi D Darmon N. High nutritional quality is not associated with low greenhouse gas emissions in self-selected diets of French adults. Am J Clin Nutr (2013) 97:569–83. 10.3945/ajcn.112.035105 23364012

[79] Drewnowski A Rehm CD Martin A Verger EO Voinnesson M Imbert P. Energy and nutrient density of foods in relation to their carbon footprint. Am J Clin Nutr (2015) 101(1):184–91. 10.3945/ajcn.114.092486 25527762

[80] Medina FX. Mediterranean diet, culture and heritage: challenges for a new conception. Public Health Nutr (2009) 12:1618–20. 10.1017/S1368980009990450 19689830

[81] Medina FX. Food consumption and civil society: Mediterranean diet as a sustainable resource for the Mediterranean area. Public Health Nutr (2011) 14(12A):2346–9. 10.1017/S1368980011002618 22166194

[82] FAO/UNEP. Sustainable Food Systems Programme. Available from: http://www.fao.org/ag/ags/sustainable-food-consumption-and-production/en/

